# Immunomodulatory behavior of CircRNAs in tumor microenvironment

**DOI:** 10.32604/or.2024.054623

**Published:** 2025-04-18

**Authors:** HAISU LIANG, WEI YAN, ZHI LIU, YUNBO HE, JIAO HU, ZHIWEI SHU, HUIHUANG LI, BELAYDI OTHMANE, WENBIAO REN, CHAO QUAN, DONGXU QIU, MINFENG CHEN, WEI XIONG, BINGNAN ZHANG, PEIHUA LIU

**Affiliations:** 1 Department of Urology, Xiangya Hospital, Central South University, Changsha, 410000, China; 2 National Clinical Research Center for Geriatric Disorders, Xiangya Hospital, Central South University, Changsha, 410000, China; 3 Department of Urology, Shimen Hospital of TCM, Changde, 415300, China; 4 Department of Urology, The Second Affiliated Hospital, Guizhou Medical University, Kaili, 556000, China; 5 NHC Key Laboratory of Carcinogenesis, Hunan Cancer Hospital and The Affiliated Cancer Hospital of Xiangya School of Medicine, Central South University, Changsha, 410013, China; 6 George Whipple Lab for Cancer Research, University of Rochester Medical Institute, Rochester, NY 14627, USA

**Keywords:** Circular RNA (circRNA), Immune response, Metabolic reprogramming, Tumor, Tumor microenvironment (TME)

## Abstract

Circular RNAs (circRNAs) are a type of non coding RNA that possess unique single stranded circular structures formed through reverse splicing mechanisms. Due to the lack of a free end that is typically susceptible to degradation by nucleases, circular RNAs exhibit resistance to ribonuclease R, making them highly stable in eukaryotic cells. The complex relationship between circRNA dysregulation and various pathophysiological conditions, especially cancer. Tumor microenvironment (TME) is a collective term for various components surrounding tumors and is an important factor affecting tumor development. Simultaneous infiltration of TME by different types of immune cells; These immune cells interact with the TME, collectively forming the so-called “tumor immune microenvironment”. The complex interactions between tumor cells and TME profoundly affect the behavior of malignant tumors, and circRNAs derived from tumor cells and TME cell components have become important mediators of immune response and evasion within the TME. CircRNAs can directly or indirectly regulate immune cells, thereby modulating anti-tumor immunity. This review highlights how circRNAs, especially those encapsulated in extracellular vesicles like exosomes, influence the differentiation, chemotaxis, and anti-tumor immune functions of immune cells within the TME. Metabolic reprogramming plays an important role in this process. The process of circRNAs regulating tumor immunity is affected by multiple factors, such as hypoxia and viral infection. This review emphasizes the roles of the interaction between circRNAs and the TME in tumor immune regulation and prospects the guiding significance of circRNAs in tumor immune checkpoint therapy.

## Introduction

Circular RNAs (circRNAs) are a distinctive class of non-coding RNAs (ncRNAs) characterized by their single-stranded cyclic structure formed through back-splicing. These molecules are notably stable in eukaryotic cells and are found across a wide range of species. Increasing evidence suggests that circRNA dysregulation is linked to numerous pathophysiological processes, such as viral infections [1], diabetes [2], and cancer [3]. The abnormally expressed circRNAs play an important role in tumor genesis, development, metastasis, and chemotherapy resistance by acting as a microRNA (miRNA) sponge, interacting with proteins and post-transcriptional regulation [4,5]. In recent years, with the efforts of researchers and the promotion of new strategies and technologies for various circRNA studies, circRNAs offer promising perspectives for cancer diagnosis and treatment.

The tumor microenvironment (TME) refers to the numerous components surrounding a tumor and is thought to be an important “hotbed” for tumor formation [6,7]. The TME is comprised of stromal cells, extracellular matrix (ECM) components, immune cells, vascular systems and various signaling entities, such as exosomes [8,9]. With the deepening of the TME studies, the importance of the TME in tumor progression and tumor immune regulation is gradually recognized, and the development of the tumor is often accompanied by changes in the TME. The TME performance and behavior may predict tumor response to immunotherapy [10,11], which contributes to predicting tumor prognosis and guiding the application of treatment regimens [12,13]. Different types of immune cells simultaneously infiltrate the TME; these immune cells interact with the TME and jointly constitute the so-called “tumor immune microenvironment,” which is an essential place for anti-tumor immunity and immune evasion [14,15]. The complex interactions between the malignant cells and the TME profoundly affect malignant tumors behavior, and circRNAs play an indispensable role in it [16,17]. CircRNAs are usually present in intracellular or extracellular vesicles, and these circRNAs are derived from tumor cells and cellular components of the TME [18,19]. CircRNAs can, directly or indirectly, affect immune cells in the TME to regulate anti-tumor immune response [20,21]. In the process of tumor development, the complex crosstalk between circRNAs and tumor cells and the TME is closely related to tumor immune regulation [16], which is affected by hypoxia and viral infection [22,23].

## Molecular Characteristics and Action Mechanism of circRNA

CircRNA, a single-stranded circular RNA molecule with a length of 100 to 1000 nucleotides, is produced by reverse splicing [24,25]. Due to the lack of a 5′ end cap structure and a 3′ end polyA tail structure, circRNA is resistant to Ribonuclease R, making it more stable [26,27].

To date, three hypothesized models of circRNA formation have been widely accepted. (1) RNA-binding proteins (RBP) mediate the formation of circRNA; the combination between RBP with intron makes the splice donor and acceptor close to each other, allowing the cyclization of extrons. Subsequently, through a series of splicing, circRNA is formed [28]; (2) The mutual pairing of introns forms circularization because hnRNA contains opposite complementary sequences and pairs on both sides to create various circRNAs [29]; (3) During the formation of mature mRNA, the exons jump, making exons close to each other, and introns get cut to form lasso cyclization, thus creating circRNA [28], the third model is the most common model of circRNAs formation.

CircRNAs can play a regulatory role through a variety of mechanisms [30,31], reviewing the latest research, we have found that they can play a regulatory role in five different ways. (1) CircRNAs can be used as a molecular sponge of miRNA, which is the most common way in the transcriptional regulation of circRNA. The expression of target genes can be increased by acting as a sponge molecule of miRNA and regulating the activity of miRNA. In bladder cancer, circ-ACVR2A serves as a sponge for miR-626, thereby influencing the expression levels of EYA4 [32]. (2) CircRNAs can also regulate gene transcription; some circRNA can regulate gene expression at the transcription level and post-transcription level, and circEIF3J and circPAPIP2 can positively regulate their gene expression by enhancing the function of transcription factors [33]. (3) CircRNAs can regulate the alternative splicing of the RNA. Alternative splicing can produce different spliceosomes in different ways, and circularized RNA can compete with linear splicing to promote abnormal transcription of oncogenes and tumor suppressor genes. For example, The circularized form of muscleblind (MBL) contains the main coding sequence and can compete with linear MBL mRNA for alternative splicing [34]. (4) CircRNAs and RNA-binding proteins can interact with each other to achieve all aspects of circRNA life cycle, such as generating, post-transcriptional regulation, translation, modification and extracellular transport [35]. (5) In addition, some circRNA has the ability to encode proteins; therefore, circRNA can serve as an important bridge between ncRNA and coding RNA [36]. Furthermore, circRNAs have the function of encoding proteins: Later studies have shown that in eukaryotes, circRNAs with internal ribosome entry sites (IRES) can effectively translate proteins [37], and circ-ZNF609 protein-coding ability provide a typical example [38].

## CircRNA and Immune Cells in TME

### CircRNA directly affects immune cells in the TME

In cancer patients, the composition of resident immune cells in the TME differs, including cytotoxic T cells, Treg cells, and tumor-associated macrophages (TAM) [39,40]. The presence of diverse immune cell populations within the tumor microenvironment correlates with the clinical prognosis of a range of cancers [41]. Tumor-infiltrating lymphocytes (TILs) exhibit a heightened specificity in their immune response directed towards neoplastic cells. In the TME, a higher abundance of TILs predicts more positive clinical outcomes for patients [42,43]. CircRNA levels in plasma exhibit a significant correlation with the abundance of TILs within the TME [42,44]. Concurrently, research has demonstrated that the expression patterns of circRNAs vary among cancer patients with high and low TIL counts, correlating with overall survival, tumor dimensions, and metastatic potential [45,46]. CircRNAs have direct or indirect effects on immune cells in the TME, which is the primary way that CircRNAs participate in tumor immune regulation. Here, we briefly review the impact of circRNA on various immune cells in the TME.

**Figure 1:**
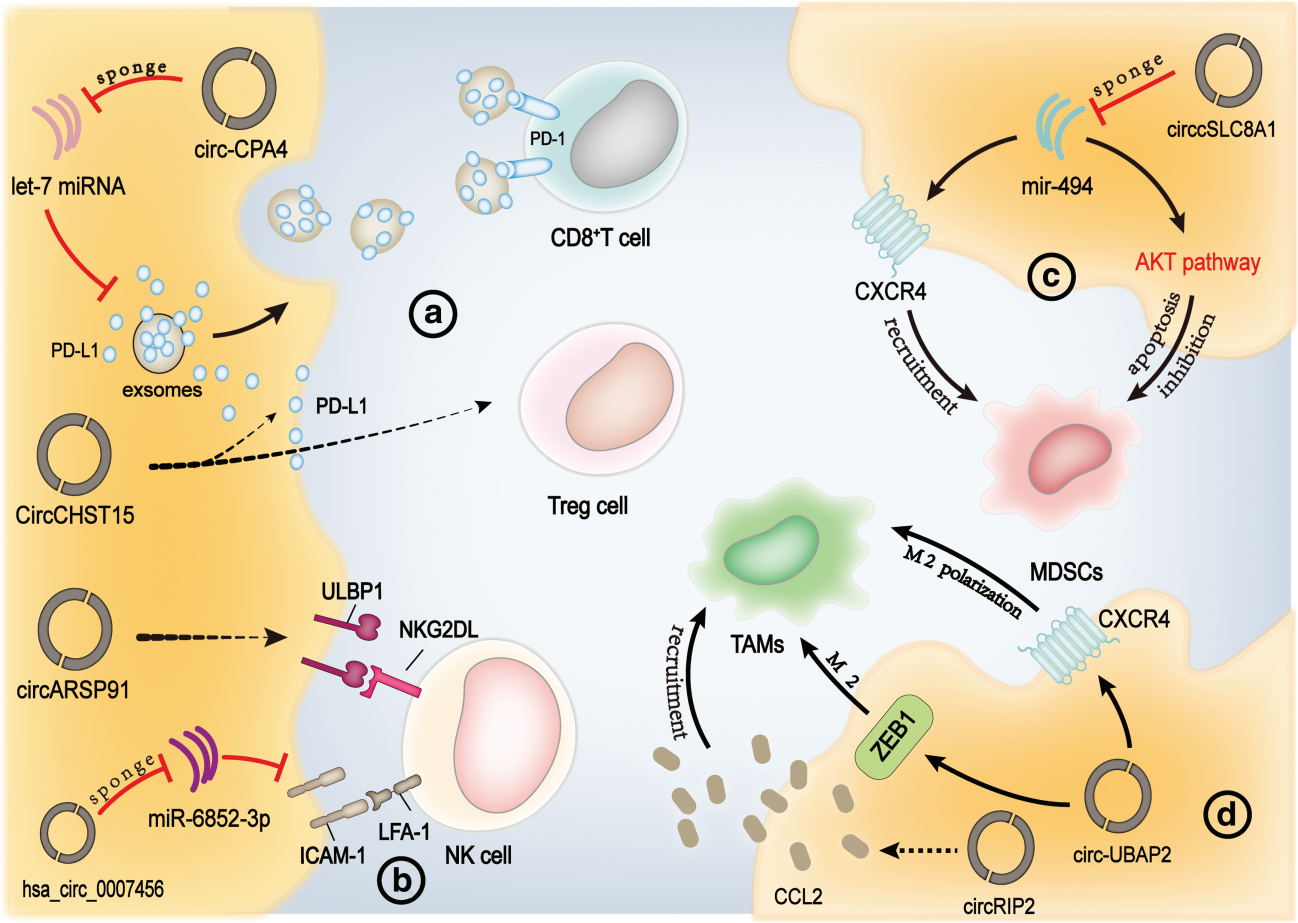
CircRNA directly affects immune cells in the TME. CircRNAs directly act on immune cells through various pathways, causing changes in immune cells, thereby altering the tumor microenvironment. ICAM-1: Intercellular adhesion molecule-1, LFA-1: Lymphocyte function-associated antigen 1, ULBP1: UL16 binding protein 1, NKG2D: Natural killer group 2D, MDSCs: Myeloid-derived suppressor cells, CCL2: Chemokine (C-C motif) ligand 2, ZEB-1: Zinc finger E-box binding homeobox 1, CCL2: C-C motif chemokine ligand 2, TAM: Tumor-associated macrophages (Figures were created using Adobe Illustrator 2022).

CD8^+^ T cells, or cytotoxic T lymphocytes (CTL) cells, are crucial in the anti-tumor immune response, killing target cells that express specific antigens. It has been found that circ-CPA4 can down-regulate let-7miRNA, which promotes tumor cells to secrete exosomal PD-L1 into the TME [47]. When PD-L1 is released into the TME, it binds to the PD-1 receptor on T lymphocytes, causing the suppression and eventual death of CD8^+^ T cells [47]. In a recent study of circRNA and PD-L1 [21], researchers got a similar result: CircCHST15 and PD-L1 were highly expressed in lung cancer, and there was a positive correlation between them. CircCHST15 was found to be targeting miR-155-5p and miR-194-5p to down-regulate their expression, and the down-regulated miRNA further inhibits PD-L1 expression in tumor tissues, At the same time, circCHST15 decreased CD8^+^ T cells in the blood and tumors of mice and increased Treg cells in mice tumors. In addition, high expression of circ_0020710 has been found to be associated with cytotoxic lymphocyte failure in the tumor microenvironment; the role and mechanism of circ_0020710 still need to be further studied [48]. More and more research suggests that circRNAs enhance the expression levels of PD-L1 by interacting with miRNAs, which in turn triggers the apoptotic process in CD8^+^ T cells [49–51] (Fig. 1a). In a study of oral squamous cell carcinoma, Circular RNA keratin 1 (CircKRT1) achieves immune evasion by dampening the cytotoxicity of CD8^+^ T cells and inducing apoptosis of CD8^+^ T cells through the CircKRT1/miR-495-3p/PDL1 axis [52]. The above studies show that the regulation of circRNA on CD8^+^ T cells in the tumor microenvironment exists objectively, but the research is not in-depth, and it is worth exploring.

Natural killer cells (NK cells), an important partin immune monitoring, are able to eliminate tumors and infected cells directly without being stimulated by either B or T lymphocytes [53,54]. However, in TME, circRNAs canaffect the function and adhesion of NK cells. NK cells trigger an immune response by recognizing Natural Killer Group 2D (NKG2D) that is expressed on the surface of viral infections or tumor cells, and UL16 binding protein 1 (ULBP1) is one of the ligands recognized by NKG2D on the surface of NK cells [55]. In hepatocellular carcinoma (HCC) cells, overexpression of circARSP91 can up-regulate the expression of ULBP1, thereby enhancing the cytotoxicity of NK cells to HCC cells [56]. Besides, recent research for HCC cell lines have found that the expression of hsa_circ_0007456 influences the susceptibility of HCC cells to NK cells. This effect is mainly achieved by controlling the expression of intercellular adhesion molecule-1 (ICAM-1) as a miR-6852-3p sponge [57]. The ICAM-1 expressed on the surface of tumor cells can be recognized by lymphocyte function-associated antigen 1 (LFA-1) on the surface of NK cells, which mediates the adhesion of NK cells to target cells and enables them to perform its immune function (Fig. 1b). In renal carcinoma (RCC), recent studies have found that circZKSCAN1 enhances the cytotoxicity of NK cells against RCC cells through the miR-1294/PIM1 axis, significantly inhibiting the proliferation and migration of RCC cells [58].

Myeloid-derived suppressor cells (MDSCs) are beneficial to tumor angiogenesis and metastasis in the TME and are famous for their ability to inhibit immune response [59,60]. A lot of non-coding RNA (ncRNA) molecules has been identified as playing an important role in the processes of proliferation and apoptosis within MDSCs in the TME [61,62]. With the further study of ncRNA, some researchers found that circcSLC8A1 participates in the above process by acting as a sponge of mir-494, affecting the migration of MDSCs to the tumor microenvironment and regulating the production of ARG1 and iNOS, thus promoting the anti-tumor immune response in the microenvironment [63]. In addition to mir-494, many kinds of microRNA have been found to be related to the proliferation and apoptosis of MDSCs in the tumor microenvironment [61] (Fig. 1c). In recent studies, investigations have revealed that the S100A9 protein, present in exosomes derived from MDSCs, upregulates the levels of circMID1. This occurs through the sequestration of miR-506-3p, thereby enhancing the expression of the MID1 gene and contributing to more rapid tumor growth [64]. Therefore, it seems beneficial for us to explore the mechanisms of circRNAs affecting immune cells infiltrated in the TME, that is, the microRNAs with known immune regulatory function may have corresponding circRNAs to affect them.

Macrophages, a vital component of innate immunity, are divided into M1 and M2 according to the catabolism of 1-arginine [65,66]. Macrophages of the M1 phenotype are known to trigger inflammatory responses, whereas their M2 counterparts exhibit anti-inflammatory properties. Examination of human cancerous tissues has revealed a significant presence of macrophages, with a notable predominance of the M2 subtype, which correlates with an unfavorable outcome in the clinical progression of various aggressive neoplasms [67,68]. Distinct expression patterns of particular circRNAs have been observed across diverse macrophage polarization states, indicating a potential significant role for circRNAs in the process of macrophage polarization [69,70]. Furthermore, recent research has indicated that elevated levels of circ-CDR1as, a circular RNA species originating from the antisense transcript of cerebellar degeneration-related protein 1 (CDR1AS), correlate with an increased prevalence of M2 macrophages. This finding implies that circ-CDR1as might participate in the polarization process of macrophages [71]. Additionally, tumor cells attract macrophages to the TME by secreting chemokines or cytokines. Upon arrival in the TME, these macrophages undergo polarization towards a TAM phenotype [72]. TAMs can not only enhance angiogenesis by secreting CXCL8 but also regulate the expression of cadherin, which promotes the migration and invasion of bladder cancer cells [73]. CircRNA has been found to regulate the malignant behavior in many tumor types by mediating the invasion of TAMs (Fig. 1d). In pancreatic adenocarcinoma (PAAD), circ-UBAP2 promotes M2 polarization of TAMs by targeting CXCR4 and ZEB1, which enhances tumor proliferation [74]. The level of circRIP2 in bladder cancer patients is correlated with clinical prognosis, and chemokine (C-C motif) ligand 2 (CCL2) is differentially expressed in circRIP2 overexpressed cells [75]. Recent research indicates that TAMs could potentially be drawn to the tumor microenvironment by the chemokine CCL2. Furthermore, these TAMs are suggested to play a role in the dissemination of bladder cancer, possibly through a complex and non-direct signaling mechanism involving circRNAs present in the tumor cells [16]. In addition, the overexpression of circASAP1 is associated with lung metastasis in patients with HCC. The molecule CircasAP1 is implicated in enhancing the growth and aggressive spread of malignant cells via interaction with the miR-326 and miR-532-5p, which in turn regulate the MAPK1 signaling cascade. Additionally, it facilitates the invasive activity orchestrated by TAMs via the same miRNAs influencing the CSF-1 signaling route [76].

The immune cells infiltrating the TME, such as dendritic cells (DCs), Treg cells and CD4^+^ T cells, have been proved to be related to the immune escape of tumor cells [77,78]. However, current studies on the relationship between circRNA and these immune cells are mainly focused on autoimmune diseases, virology and other fields. Therefore, it has not been summarized in this review, but relevant research results are expected to emerge in the future to improve further the immune regulatory network of circRNAs in the TME. By summarizing the above direct regulation of circRNA on immune cells, we found that circRNA is involved in differentiation, chemotaxis, proliferation, apoptosis and anti-tumor immune function of infiltrating immune cells in the TME, but further research needs to be deepened and expanded. In addition to influencing immune cells in the tumor microenvironment, circRNA may also build an immunosuppressive microenvironment for tumor cells by regulating cytokines and chemokines to promote tumor immune escape [79]. Further research is necessary to clarify the mechanisms in detail.

### CircRNAs induce metabolic competition between tumor cells and immune cells

In recent years, many studies have found that tumor progression is accompanied by changes in tumor cell metabolic profile and abnormal activation of metabolic pathways, which provide a material basis for the rapid proliferation of tumor cells and help tumor cells resist cell death signals [80,81]. At the same time, the metabolism of immune cells is of great significance. Metabolic reprogramming of immune cells and the TME changes caused by tumor cell metabolism both affect the function of immune cells [53]. Metabolic reprogramming of tumor cells affects anti-tumor immune activities in the TME through various mechanisms, such as enhancing the function of inhibitory immune cells and interfering with the metabolism of other cells in the microenvironment, thus forming an immunosuppressive microenvironment [82,83]. Specifically, tumor cells that undergo metabolic reprogramming compete with immune cells in the TME for nutrients. In the process of competing with tumor cells with high metabolism, the regular activities of the immune cells will get interfered with, and the anti-tumor immune function will be inhibited [84]. Concurrently, metabolites secreted by tumors within the TME have the capacity to modulate the differentiation and functional capabilities of immune cells [85]. CircRNAs in the TME affect tumor immunity by orchestrating the metabolic reconfiguration of neoplastic cells and the metabolic governance of immune cells, thereby serving as a pivotal link between metabolic processes and immune reactions [86]. Studies have found that circRNA inhibits the anti-tumor immune activity of immune cells and promotes tumor immune evasion through metabolic competition between tumor cells and TME infiltrating immune cells [84].

To meet the increased demand for macromolecule synthesis under hypoxic conditions, many tumor cells undergo metabolic reprogramming to grow rapidly. These tumor cells require a higher rate of glycolysis to maintain rapid growth. Meanwhile, glycolysis is crucial for the proliferation and function of activated T cells. Many studies have shown that the glycolysis activity of tumor cells, mediated by circRNA, limits the glucose consumption of TILs leading to T cells failure and immune escape, and that metabolic limitation of T cell activation, inhibition of mTOR activity, reduction of glycolysis and IFN-y production, leads to tumor progression [87,88]. CircRNA promotes the glycolysis in tumor cells to inhibit T cells in TME. Enolase 1 (ENO1), a glycolytic enzyme, promotes tumor progression in some tumor types, including colorectal cancer [89], non-small cell lung cancer [90], and breast cancer [91]. The researchers found that circ-ENO1 and its host gene ENO1 were up-regulated in lung adenocarcinoma (LUAD) cells. Circ-ENO1, like a sponge, interacts with miR-22-3p to up-regulate the expression of ENO1 and promote glycolysis in LUAD cells. The vivo experiments further confirmed that circ-ENO1 promoted tumor growth and metastasis [92]. The increased glycolytic activity in tumor cells is often associated with hypoxic conditions within the tumor microenvironment, complementary to the rise of glycolysis of tumor cells. When hypoxia occurs, CircMAT2B leads to higher expression of the target gene PKM2 as a sponge for miR-338-3p. PKM2, being an important enzyme involved in glycolysis, pushes glycolysis in tumor cells. This higher level of glycolytic activity is a contributing factor to the development of liver cancer in patients [84]. Similarly, under hypoxia, knocking down circDENND4C can inhibit glycolysis of breast cancer by up-regulating miR-200b/c [93].

**Figure 2:**
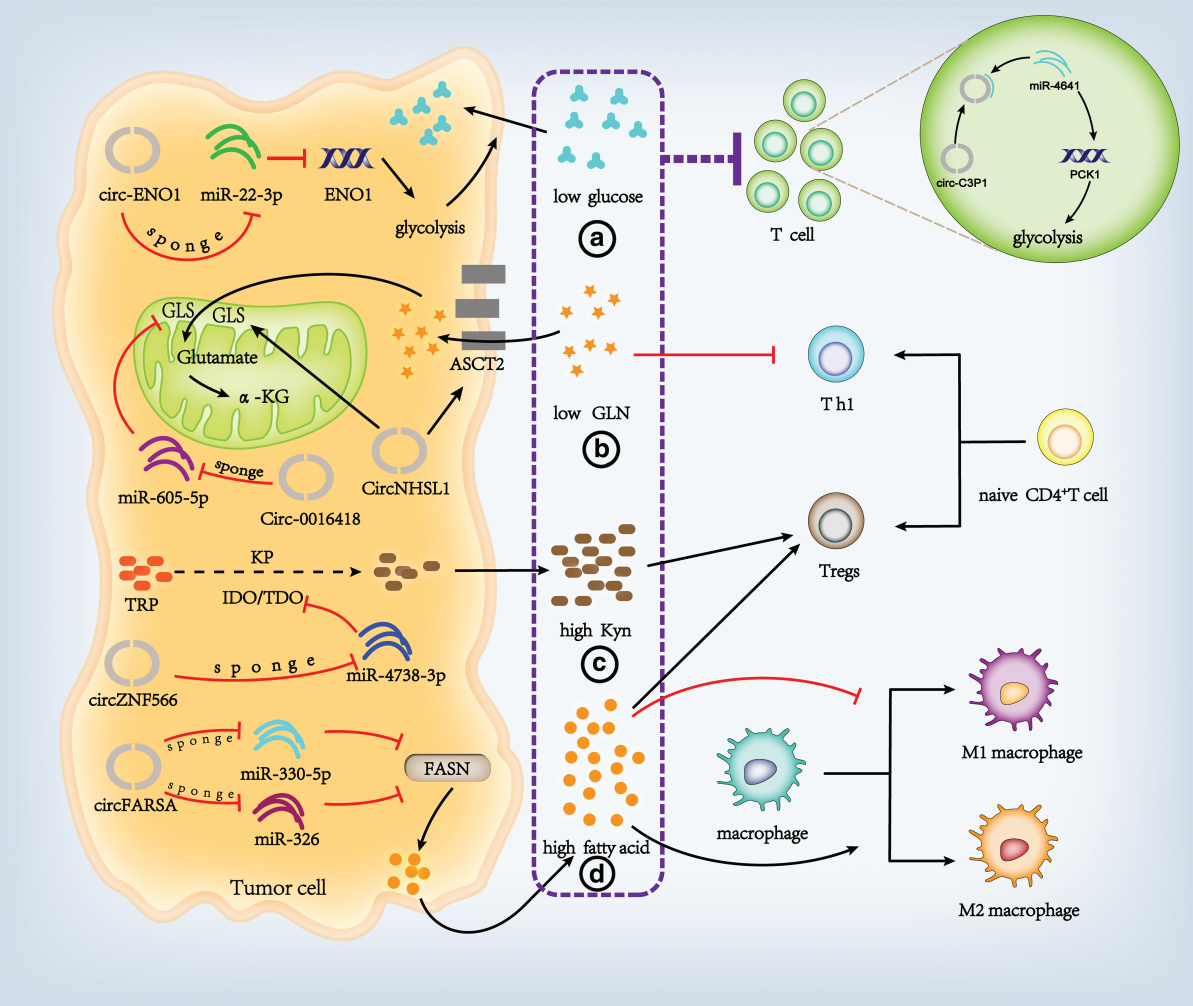
CircRNAs induce metabolic competition between tumor cells and immune cells. CircRNAs can induce metabolic competition between tumor cells and immune cells through different pathways. GLS: Glutaminase, α-KG: α-ketoglutaric acid, KP: Kynurenine pathway, IDO: Indoleamine-2,3 dioxygenase, TDO: Tryptophan-2,3 dioxygenase, FASN: Fatty acid synthase, GLN: Glutamine, Kyn: Kynurenine (Figures were created using Adobe Illustrator 2022).

CircRNAs are involved in the glycolytic reprogramming of tumor cells to affect metabolic competition within the TME and inhibit immune cell function. However, circRNAs can also counter this inhibition by increasing the glycolysis capacity of immune cells. It has been found that overexpression of PCK-1 in T cells can promote glycolysis and restore the anti-tumor effect of T cells [87]. CircC3P1 can promote the expression of PCK1 in HCC cells by acting as a mir-4641 sponge, which can significantly weaken the proliferation, migration and invasion activities of HCC cells [94]. By contralling the glycolytic activity of T cells via PCK1, circRNA can alter the metabolic competition that occurs between tumor cells and T cells within the tumor microenvironment (Fig. 2a).

Beyond glycolysis, the breakdown of glutamine represents a significant indicator of metabolic reconfiguration in cancer cells. In T cells, glutamine regulates mTOR activation and serves as a key substrate for the synthesis of proteins that govern effector T cell activity and differentiation [95]. Blocking glutamine uptake in CD4^+^ T cells could inhibit the production of Th1 cells but did not affect the differentiation of Tregs [96]. Some scholars believe that similar to the glycolysis competition, the decrease of glutamine in TME may have a negative effect on the function of immune cells [85]. Under physiological conditions, glutamine enters the cell through transporters such as LAT1 and ASCT2 and is subsequently converted to glutamate by glutaminase (GLS), glutamine dehydrogenase (GDH) and other enzymes. Next, it gets converted to α-ketoglutaric acid (α-KG) [97] and then participates in a cellular biochemical reaction. In 2019, researchers found that CircHMGCS1 can promote glutamine decomposition in hepatoblastoma cells [98]. In 2020, additional studies reported that the down-regulation of circNHSL1 led to a decrease in the expression of glutamine, glutamate and α-KG in gastric cancer cells due to the knockout of circNHSL1, which suppresses the protein levels of ASCT2 and GLS [99]. Therefore, circNHSL1 knockdown inhibits glutamine uptake by gastric cancer cells in TME, inhibits tumor growth *in vivo*, and reduces glutamine competition with CD4^+^ T cells [99]. Another project team reported their findings that Circ_0016418 could act as a miR-605-5p sponge to remove the inhibition of miR-605-5p on GLS, increase cell uptake, transform glutamine and promote the development of melanoma [100]. MiRNA can regulate gene expression by pairing with the bases of cytoplasmic mRNA 3′ UTR (Fig. 2b). The inhibitory effect of miR-605-5p on GLS is to bind directly to GLS-3 UTR and negatively regulate the expression of GLS. CircRNA promotes glutamine metabolism of tumor cells, and competition inhibits the function of T cells in TME. However, it does not affect the differentiation of Tregs, which makes tumors proliferate and progress rapidly and, most importantly, be prone to immune escape.

Tumor cells and cancer-related fibroblasts can lead to immunosuppression by metabolizing tryptophan to kynurenine [101,102]. The kynurenine pathway (KP) is the pathway by which tryptophan is degraded to kynurenine. The speed of KP is limited by tryptophan-2,3 dioxygenase (TDO2) and indoleamine-2,3 dioxygenase (IDO) in the liver. Various pro-inflammatory cytokines, such as IL-1, IFN-γ and TNF α, can up-regulate the IDO expression, increasing kynurenine produced by tumors [101,102]. Within the TME, kynurenine engages the aromatic hydrocarbon receptor (AhR) on T cells, promoting the activation and transformation of regulatory Tregs. Additionally, kynurenine directly inhibits effector T cell function by blocking IL-2 signaling, which leads to a reduction in CD4^+^ memory T cells [103]. CircRNA was also found to be involved in the above tryptophan metabolism; CircZNF566, as a miR-4738-3p sponge, alleviates its inhibition on TDO2 [104]. Although this study did not further explore the effect of circZNF566 on tryptophan metabolism, it identified that circRNA participates in the tryptophan metabolism regulation (Fig. 2c).

Lipids are one of the necessary components of cell composition, and the swift proliferation of cancer cells is inseparable from the supply of fatty acids. It has been found that, unlike normal tissues, which are more dependent on exogenous fatty acids, tumor tissues can synthesize fatty acids themselves [105], cancer cells produce large amounts of fatty acids through the reprogramming of fatty acid metabolism to meet the lipid demand for rapid proliferation [106,107]. And the production, storage and transport of these fatty acids affect the rapid proliferation and malignancy of tumor cells. Fat-rich microenvironments suppress the activity of effector T cells and the M1 polarization of macrophages, concurrently promoting the differentiation of Tregs and M2-like macrophages [108]. Extensive research has validated the involvement of circRNAs in the processes of adipose tissue development, lipolysis, and the modulation of adipose tissue differentiation and transformation [109]. Additionally, circRNAs are recognized for their significant impact on the reprogramming of fatty acid metabolism in cancer cells [110]. Fatty acid synthase (FASN) is a critical metabolic multi-enzyme in fatty acid synthesis; the up-regulation of fatty acid synthesis caused by the overactivity and expression of FASN provides a nutritional basis for the generation, proliferation and invasion of tumor cells [111]. As a sponge of miR-330-5p and miR-326, circFARSA alleviated the inhibition of FASN and promoted the lipid metabolism of tumor cells [112] (Fig. 2d).

CircRNAs affect the concentration and distribution of key nutrients in the TME through the metabolic reprogramming of tumor cells so that the differentiation and function of immune cells infiltrating the TME can be changed in favor of tumor escape. To counteract this change, circRNAs can also influence immune cells to adapt to the metabolic competition. Understanding the role of circRNAs in metabolic competition between tumor cells and immune cells will help us further understand the immune regulation mechanism of circRNAs in the TME and provide guidance for targeted therapy of tumors. Studies on circRNAs in the TME that influence tumor immunity through metabolic reprogramming mainly focus on glycolysis; but We expect the performance of circRNAs in other metabolic pathways and hope that the research results can be applied clinically as soon as possible.

## Hypoxia and Viral Infection Influence the Immune Regulatory Behavior of circRNAs in the TME

### Hypoxia-induced regulation of the TME by circRNAs

Hypoxia is a common feature of solid tumors and is related to tumor invasiveness and poor prognosis. Lactic acid produced by hypoxia reduces the expression of autophagy factor FIP200 and induces apoptosis of natural T cells [113]. Lactic acid also inhibits T cell movement and inhibits cytotoxicity and effector functions [114]. It can also promote the polarization of macrophages to M2 by stabilizing HIF-1A [115]. Hypoxia can induce the expression of key circRNAs. As a miRNA sponge, circRNA activates the translation of hypoxia-related factors such as HIF1-α through the circRNA-miRNA-mRNA axis, which influences the activity and functionality of immune cells within the tumor microenvironment, thereby potentially fostering the progression of cancer growth.

**Figure 3:**
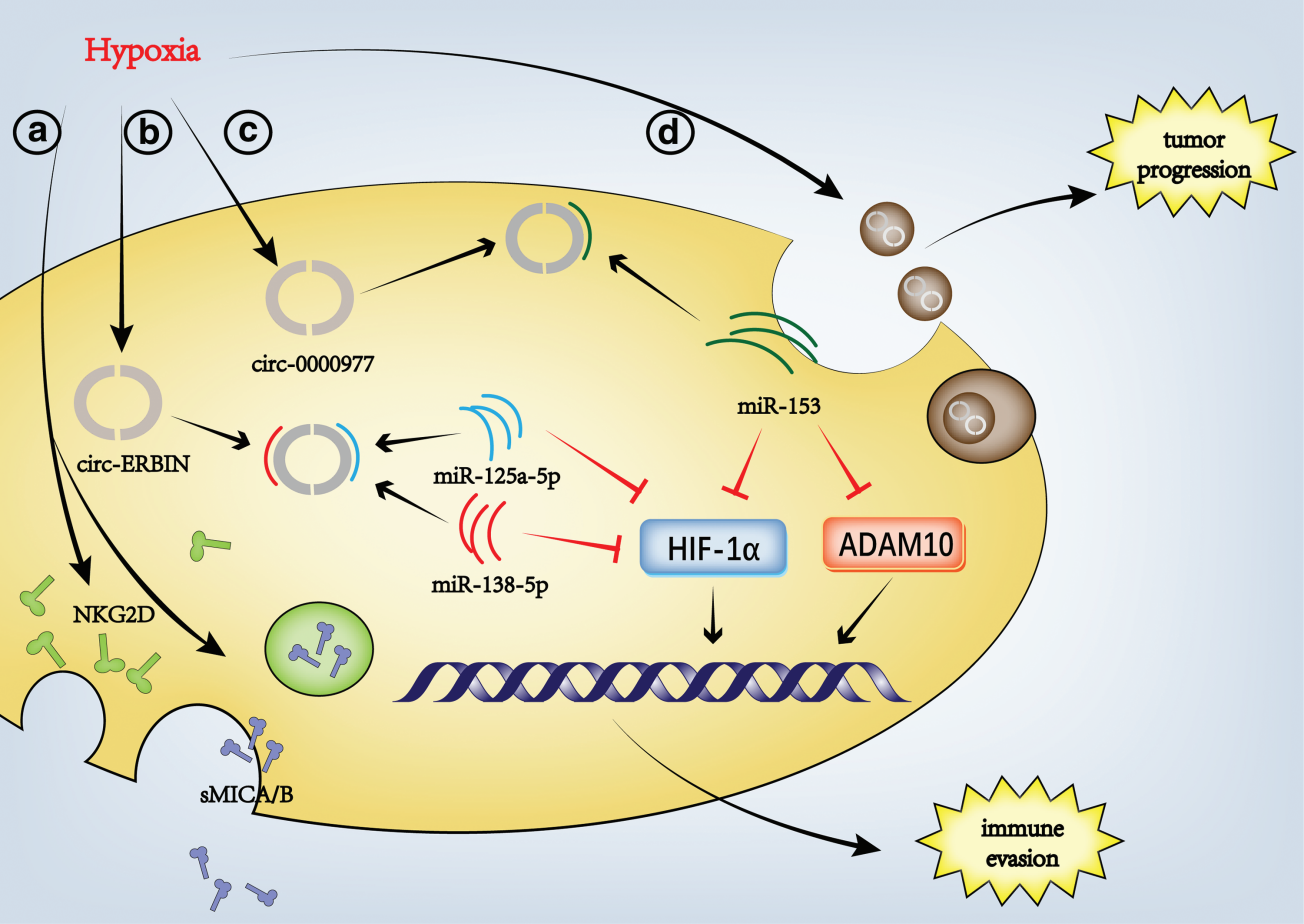
Hypoxia-induced regulation of the TME by circRNAs. Hypoxia can induce the impact of circRNA on the tumor microenvironment through various pathways. NKG2D: Natural killer group 2D, sMICA/B: Soluble major histocompatibility complex class I Chain-related A/B, HIF-1α: Hypoxia-inducible factor 1-α, ADAM10: A disintegrin and metalloproteinase 10 (Figures were created using Adobe Illustrator 2022).

One of the keys to the malignant progression of pancreatic cancer (PC) is that tumor cells acquire the ability to escape immune-mediated cytolysis. The hypoxic microenvironment significantly contributes to the metastatic spread of PC, enabling tumor cells to escape from the immune system’s surveillance. In NK cells with high HIF1-α expression, the internalization levels of MICA/B and NKG2D were significantly higher than those of NK cells with low HIF1-α expression. Hypoxia considerably increased the supernatant level of PANC-1 cell sMICA culture. Hypoxia can stimulate the up-regulation of HIF1A, ADAM10, and sMICA, resulting in the reduction of NKG2D in NK cells and tumor cells and evade immune surveillance and NK cell-mediated lysis [116] (Fig. 3a). CircRNA plays a vital role in the process of hypoxia-induced tumor immune escape. Hypoxia induces the expression of CIRC_0000977 (one kind of circRNAs), and the knockdown of CIRC_0000977 enhances the killing effect of NK cells on hypoxia PC cells by HIF1-α and ADAM10 [116]. HIF1-α and ADAM10 are identified as the immediate downstream effectors of miR-153. The circular RNA CIRC_0000977 functions as a molecular sponge for miR-153, neutralizing the inhibitory impact of miR-153 on the expression of HIF1 and ADAM10 mRNAs by specifically engaging with 293T and Panc-1 cell lines. It has been demonstrated that circ-ERBIN enhances HIF-1α transcription and promotes growth and metastasis in colorectal cancer, a process in which HIF is up-regulated via two microRNAs (miR-125a-5p and miR-138-5p/4eBP-1). At the same time, circerbin can promote tumor angiogenesis [117] (Fig. 3b). Conversely, the suppression of miR-153 activity results in an opposing effect on the immune evasion facilitated by HIF1A in PC cells, which is induced by the knockdown of circ_0000977. The effect of circ_0000977 knockout was partially reduced by miR-153 inhibition [116] (Fig. 3c).

In addition, the hypoxic TME seems to promote tumor progression by secreting exosomes with specific circRNAs. For example, Stimulated by hypoxia, multiple myeloma cells released more exosomes whose ncRNA could enhance angiogenesis [118]. Another study indicates that under hypoxic conditions, circPRDM4 facilitates the recruitment of hypoxia-inducible factor-1α (HIF-1α) to the promoter of CD274, thereby inhibiting the infiltration of CD8 T cells in the tumor microenvironment and promoting the immune evasion of hepatocellular carcinoma cells [119] (Fig. 3). Unfortunately, there is no study researching the effects of tumor cell-derived exosomes induced by hypoxia on immune cells, which may be a promising research field.

### Viral infection induces immune regulation of the TME by circRNAs

Virus infection is one of the initiating factors of a malignant tumor, and the chronic infection of many viruses can transform normal cells into malignant cells [120]. In the process of the occurrence and development of these tumors, viruses not only act as the initiating factor but also participate in the immune escape of tumors. Both endogenous circRNAs and virus-encoded circRNAs are involved in the above process.

Persistent infection with the hepatitis B virus (HBV) is a major contributor to the development of hepatocellular carcinoma. 90% of infected infants and 5%–10% of healthy adults develop chronic hepatitis B (CHB) [121]. CircRNAs are crucial in influencing the development and advancement of CHB [122,123]. Follistatin-like protein 1 (FSTL1) is associated with many diseases, especially fibrosis and tumorigenesis [124]. In tumors, FSTL1 can affect immune cell infiltration in the TME, participate in the activation of dendritic cells and T-lymphocyte, and enable tumors to escape immune surveillance [125,126]. Zhang et al. found that circ_0004812 is upregulated in CHB and modulates HBV-induced immunosuppression by promoting FSTL1 expression, which is done through inhibiting mir-1287-5p [23].

Viruses can encode circRNAs in somatic cells and malignancies, and these circRNAs are involved in the process of tumor deterioration, proliferation, invasion and metastasis [127–129]. Recent studies have found that virus-encoded circRNA is involved in the immune regulation of tumors in the TME. Infection by the Epstein-Barr virus (EBV) is recognized as a primary etiological factor in the development of nasopharyngeal carcinoma (NPC) and contributes to a spectrum of malignant characteristics [120]. In NPC, EBVs can encode multiple circRNAs. High expression of EBV-encoded circBART2.2 promotes PD-L1 transcription, inhibiting T cell function in the TME and leading to tumor immune escape [22].

The mechanism of viral infection initiating tumorigenesis has been of continuing interest, but the involvement of endogenous circRNAs and virus-encoded circRNAs in immune regulation of virus-associated tumors has only been studied over the last two years. And we believe that more circRNAs that regulate virus-associated tumor immunity will be discovered in the future.

## Exosomal circRNA Mediated Immune Regulation of the TME

In the TME, intercellular communication profoundly affects tumor progression and immune response, partially mediated by exosomes derived from different cell types [16,130]. Moreover, intercellular exosome is a key to exploring further the role of circRNAs in immune regulation in the TME [131,132]. The expression profile of the exosomal circRNA was different from that in parent-cell, indicating that circRNA was actively incorporated into exosomes [133,134]. However, the exact mechanism by which circRNA enters exosomes remains unclear.

**Figure 4:**
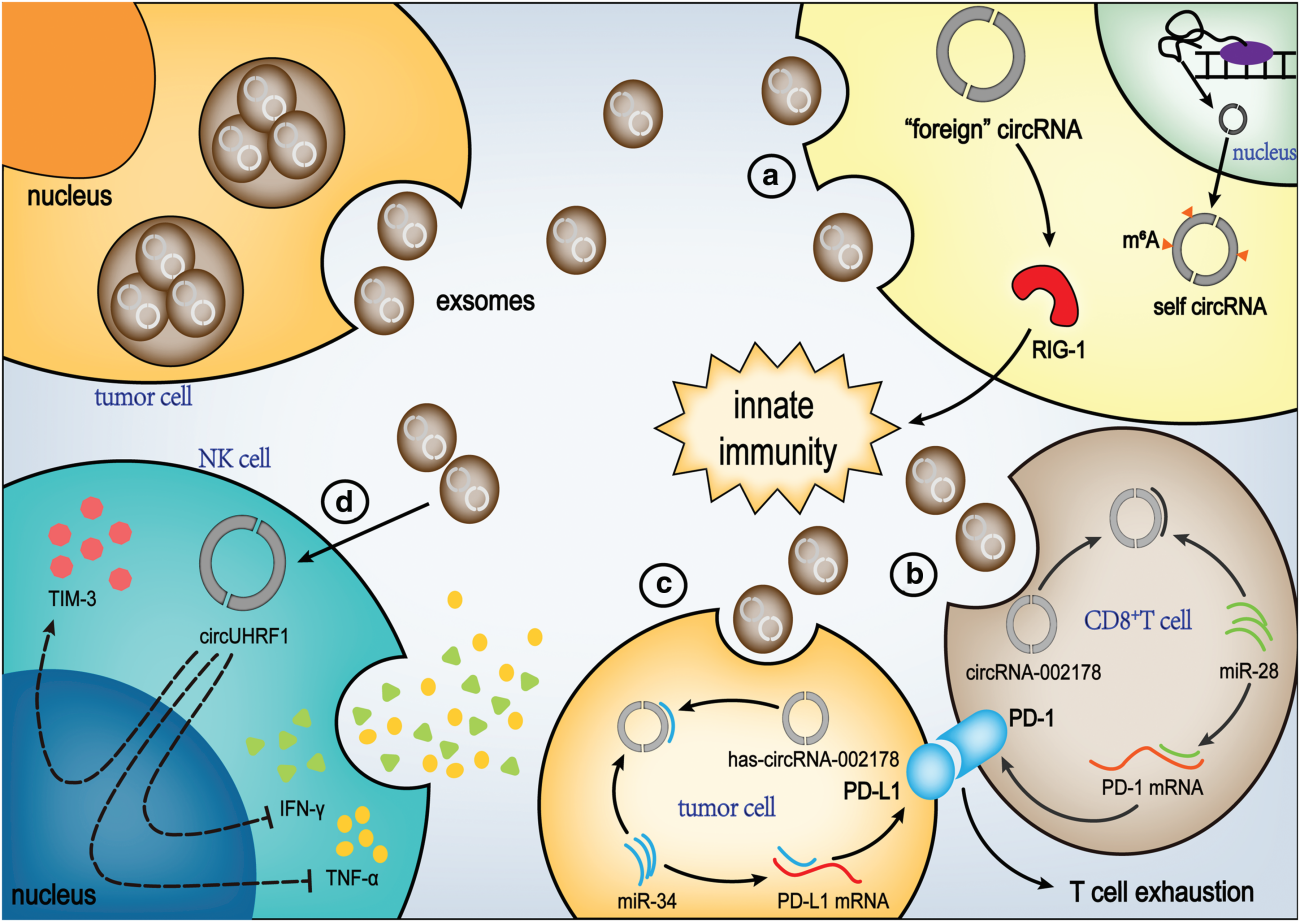
Exosomal circRNA mediated immune regulation of the TME. Exosomal circRNA can influence the tumor microenvironment through various immune mechanisms. NK cell: Natural killer cell, TIM-3: T cell immunoglobulin domain and mucin domain-3, RIG-1: Retinoid-induced gene 1, IFN-γ: Interferon-γ, TNF-α: Tumor necrosis factor-α, PD-1: Programmed death-1 (Figures were created using Adobe Illustrator 2022).

Tumor-derived exosomes are enriched with antigens originating from the malignancy, capable of being intercepted by dendritic cells. This interaction facilitates the display of these tumor-specific antigens, which subsequently triggers an immune response that targets the cancerous cells. Nonetheless, these exosomes are also laden with an array of immunosuppressive factors. These factors have the potential to deactivate T lymphocytes or NK cells, and they can also foster the development of Tregs or MDSCs, both of which are known to dampen the immune system’s reaction to the tumor. Newly generated circRNAs in tumors can be encapsulated within exosomes for transport [135], and they are delivered to immune cells as a tumor antigen to activate anti-tumor immunity or be combined with miRNAs [136] and proteins to regulate immune cell activity [137]. Functioning as antigens within the immune system, exogenously isolated circRNAs could potentially stimulate the innate immune response *in vitro*. This process may occur via the activation of the retinoid-induced gene I (RIG-I) signaling pathway [137]. CircRNAs can trigger the RIG-I nucleic acid sensor, which is recognized for its role in modulating innate immunity. Agonists targeting RIG-I have demonstrated the ability to stimulate immune responses that combat cancer cells within tumors [138,139]. Therefore, exogenous circRNAs (“foreign” circRNAs) entering tumor cells can potentially affect RIG-I and activate anti-tumor immunity. However, RIG-I is not activated by circRNAs modified by N^6^-methyladenosine (m^6^A) [140]. As an RNA modification, m^6^A isolates and blocks endogenous circRNA activation of RIG-I anti-tumor pathways. Thus, m^6^A modification may help the tumor escape immune surveillance. Circe7 modified by m^6^A, for example, can promote the growth of CASKI cervical cancer cells *in vivo* [127] (Fig. 4a).

In addition, circRNA secreted from tumor cells can act in recipient cells, including immune cells, leading to immune escape of the tumor and malignant transformation of normal cells [130,141]. For example, studies have shown that exosome-derived circATP8A1 from gastric cancer cells induces M2 polarization of macrophages and tumor progression through the circATP8A1/miR-1-3p/STAT6 axis [142].

The regulatory effect of circRNAs on immune cells has long been established. This regulatory effect is displayed when tumor cells indirectly regulate the immune system in TME through tumor exosomes packaged with circRNAs, which is an important means of immune escape for tumorogenesis. CircRNA-002178 can enter CD8^+^ T cells via exosomes and act as a miR-28-5p sponge to induce PD-1 expression [143]. Previous research has demonstrated that miR-28-5p suppresses the expression of PD-1 by directly interacting with the 3′ UTR of the PD-1 gene in CD8^+^ T lymphocytes [144] (Fig. 4b). In addition, HAS-circRNA-002178 can enhance the expression of PD-L1 in cancer cells by miR-34 and induce T cell failure [143](Fig. 4c). Zhang et al. reported their research in 2020 that HCC cell exosomes mainly secreted circUHRF1 in the plasma of HCC patients, and the circUHRF1 inhibited the secretion of NK cell-derived IFN-γ and TNF-α. Elevated concentrations of circUHRF1 within plasma exosomes correlate with a decrease in the proportion of NK cells and a diminished infiltration of NK cells into tumor tissues. (Fig. 4d) [20]. In addition, circUHRF1 inhibited NK cell function by degrading miR-449C-5p and up-regulating the expression of Tim-3 [145]. Also, circUHRF1 may have caused HCC patients to develop resistance to PD1 immunotherapy [20].

These findings suggest that circRNAs may be transported through exosomes to the immune cells, which may either induce immune response as a tumor antigen or impair the function of immune cells.

## CircRNAs and Immune Checkpoint Inhibition Therapy

The primary mechanism by which tumors evade immune detection involves the establishment of an immunosuppressive milieu, a state that can potentially be reversed through the application of immune checkpoint blockade therapies. Tumor cells can silence immune responses by overexpressing “immune checkpoint” molecules. For example, the combination of PD-L1 overexpressed on tumor cells and PD-1 on T cells can promote T cell apoptosis and induce Tregs differentiation [146]. At present, immune checkpoint inhibitor therapy targeting PD-1 and PD-L1 has been applied in clinical practice, but the overall efficiency of immune checkpoint inhibitor therapy is still not satisfactory [147]. As mentioned above, circRNAs can affect the expression of PD1/PDL1, leading to the depletion of anti-tumor immune cells and the formation of immunosuppressive microenvironment. For example, CircCHST15 inhibits the expression of PD-L1 in tumor cells [21], and the up-regulation of circ-CPA4 enables tumor cells to deliver PD-L1 to tumor microenvironment through exosomes [47]. In addition to enhancing PD-L1 expression in tumor cells, circrNA-002178 can also induce PD-1 up-regulation through exosome transfer to CD8^+^ T cells [143]. In addition to direct regulation of PD-1/PD-L1, circRNAs are also associated with drug resistance to immune checkpoint therapy. CircDLG1 regulates CXCL12 by sponging Mir-141-3p to promote gastric cancer progression and anti-PD-1 resistance [148]. Due to the critical role of circRNAs in regulating the PD-1/PD-L1 pathway, these circRNAs are considered as adjuvant immunotherapy targets to improve the efficiency of immune checkpoint inhibition therapy in cancer patients. Another immune checkpoint that attracts attention is CTLA-4 [149]. However, until now, no convincing evidence has been found on whether circRNAs affect or regulate CTLA-4. We expect new achievements and breakthroughs in this field.

## Conclusion

CircRNAs play an important role in immune regulation in the TME. CircRNAs can directly affect the differentiation, chemotaxis, proliferation, apoptosis and anti-tumor immune function of infiltrating immune cells in the TME, enabling tumor cells in the TME to escape immune surveillance. On the other hand, circRNA affects the concentration and distribution of critical nutrients in the TME by participating in the metabolic reprogramming of tumor cells, which causes metabolic competition between tumor cells and immune cells, ultimately leading to changes in the differentiation and function of immune cells that are conducive to tumor escape from immune monitoring. In addition to promoting tumor progression, hypoxia and viral infection also regulate tumor immunity through circRNAs. Hypoxia is a common characteristic of solid tumors, and viral infection is one of the initiating factors of malignant tumors. Studying the changes of circRNAs in the TME under these two conditions can help develop specific therapeutic methods and preventive measures for tumors. At the same time, under the regulation of exosomes as a communication medium, circRNA affects tumor progression through immune regulation in the TME with a complex and tight network. Exosomal circRNAs affect the progression of tumors through mechanisms such as immune cell activation, enhancement of anti-tumor immune responses, metabolic reprogramming, and regulation of immune checkpoint molecules, indicating their great potential in the field of cancer immunotherapy. This review summarizes the immunological functions of circRNA in the tumor microenvironment, but due to the continuous conduct of related experiments, the review may have omitted some studies. The current research on circRNA and its immune regulatory functions has found that the direct impact on various immune cells in TME is not yet thoroughly studied. In addition, although metabolic competition is an important research field, most studies focus on glycolysis and explore less the role of circRNAs in other metabolic pathways.

Although there is increasing body of research indicates that circRNAs play an immunomodulatory role in the TME, the research in this field is still in its infancy, and it will take a long time and much work to truly reveal their internal mechanisms. In terms of circRNAs therapy, although no clinical trials have been reported so far, we believe that circRNAs can be used clinically to regulate the abundance or activity of immune cells and for early detection, diagnosis and treatment of cancer in the near future. Future studies will require a significant number of prospective, large-sample, multi-center clinical trials to validate the changes of circRNAs in the tumor microenvironment of cancer patients, in order to develop corresponding clinical diagnostic markers or related immunotherapies.

## Data Availability

Not applicable.

## Abbreviations

TME: Tumor microenvironment
ECM: Extracellular matrix
RBP: RNA-binding proteins
MBL: Muscleblind
IRES: Internal ribosome entry sites
TAM: Tumor-associated macrophages
TILs: Tumor-infiltrating lymphocytes
CTL: Cytotoxic T lymphocytes
NKG2D: Natural Killer Group 2D
ULBP1: UL16 binding protein 1
HCC: Hepatocellular carcinoma
ICAM-1: Intercellular adhesion molecule-1
LFA-1: Lymphocyte function-associated antigen 1
MDSCs: Myeloid-derived suppressor cells
PTEN: Phosphatase and Tensin Homolog
PAAD: Pancreatic adenocarcinoma
CCL2: Chemokine (C-C motif) ligand 2
DCs: Dendritic cells
GLS: Glutaminase
GDH: Glutamine dehydrogenase
α-KG: α-ketoglutaric acid
KP: Kynurenine pathway
TDO2: Tryptophan-2: 3 dioxygenase
IDO: Indoleamine-2: 3 dioxygenase
AhR: Aromatic hydrocarbon receptor
PC: Pancreatic cancer
CHB: Chronic hepatitis B
FSTL1: Follistatin-like protein 1
EBV: Epstein Barr virus
NPC: Nasopharyngeal carcinoma
RIG-I: Retinoid-induced gene I
